# Supplementary material for: The causal role of circulating inflammatory markers in osteoporosis: a bidirectional Mendelian randomized study
Source: Front Immunol. 2024 Jul 18;15:1412298. doi: 10.3389/fimmu.2024.1412298 (PMC11291241; doi:10.3389/fimmu.2024.1412298)
Supplement: Supplementary file 1 [file DataSheet_1.zip › supplementary material/CIM-OSTEOPOROSIS /GCST90274829.csv_forest.pdf]

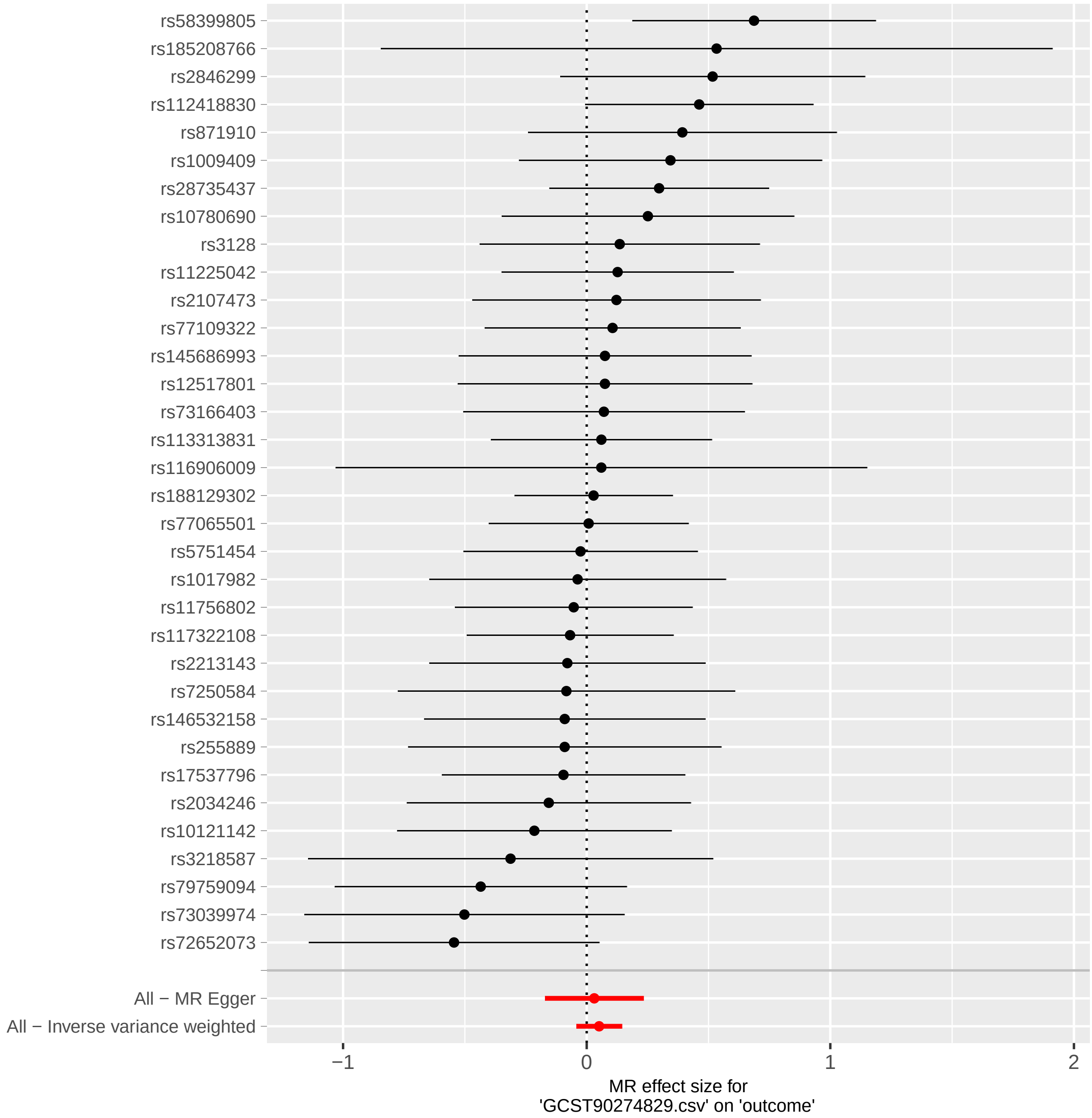
rs58399805
rs185208766
rs2846299
rs112418830
rs871910
rs1009409
rs28735437
rs10780690
rs3128
rs11225042
rs2107473
rs77109322
rs145686993
rs12517801
rs73166403
rs113313831
rs116906009
rs188129302
rs77065501
rs5751454
rs1017982
rs11756802
rs117322108
rs2213143
rs7250584
rs146532158
rs255889
rs17537796
rs2034246
rs10121142
rs3218587
rs79759094
rs73039974
rs72652073
All − MR Egger
All − Inverse variance weighted
−1
0
1
2
MR effect size for
'GCST90274829.csv' on 'outcome'
